# Extracellular Polysaccharide Extraction from *Streptococcus thermophilus* in Fermented Milk

**DOI:** 10.1128/spectrum.02280-21

**Published:** 2022-03-28

**Authors:** Yunchao Wa, Ryan Matthew Chanyi, Hanh Thi Hong Nguyen, Ruixia Gu, Li Day, Eric Altermann

**Affiliations:** a Jiangsu Key Laboratory of Dairy Biotechnology and Safety Control, Yangzhou University, Yangzhou, People’s Republic of China; b College of Animal Science and Technology, Yangzhou University, Yangzhou, People’s Republic of China; c College of Food Science and Engineering, Yangzhou University, Yangzhou, People’s Republic of China; d Grasslands Research Centre, Te Ohu Rangahau Kai, AgResearch, Palmerston North, New Zealand; e Riddet Institute, Massey University, Palmerston North, New Zealand; University of Torino

**Keywords:** extracellular polysaccharide, *Streptococcus thermophilus*, dairy, fermentation

## Abstract

Lactic acid bacteria such as Streptococcus thermophilus are known to produce extracellular polysaccharide (EPS) in fermented foods that enhance the creaminess and mouthfeel of the product, such as yogurt. Strains producing larger amounts of EPS are highly sought-after, and therefore, robust and accurate quantification methodologies are important. This study found that two commonly used methodologies significantly underestimated the amount of EPS produced as measured using a milk matrix. To this end, a proteolytic step was implemented prior to EPS extraction (Method C). An initial proteolytic step using xanthan gum-spiked milk significantly increased recovery yield to 64%, compared to 27.8% for Method A and 34.3% for Method B. Method C showed no improvement when assessed using a chemically defined medium. Method C was further validated using three strains of S. thermophilus with varying EPS-production capabilities (ST^LOW^, ST^MID^, ST^HIGH^). Overall, Method C demonstrated significant improvements in the EPS extraction yield for all three S. thermophilus strains in fermented milk. On average, Method C improved isolation yield by ∼3- to 6-fold compared with Method A and by ∼2- to 3-fold compared with method B. There were no significant differences between samples when they were grown in a chemically defined medium, highlighting the importance of a proteolytic step specifically for fermented milk samples. In commercial applications, accurate quantification of EPS-production is an important aspect when finding new strains.

**IMPORTANCE** Extracellular polysaccharide (EPS) production by milk-fermenting microorganisms is a highly sought-after trait in improving the perceived thickness, creaminess, and mouthfeel of yogurt. Streptococcus thermophilus are commonly isolated and their EPS production is quantified in the search for higher-producing strains. In this study, we demonstrated that two commonly used methods for isolating EPS from milk samples significantly underestimated the true amount of EPS present. We demonstrated that the addition of a proteolytic step prior to EPS extraction isolated over 2-fold more EPS than identical samples processed using the traditional protocols. We further validated this method in fermented milk samples from three strains of S. thermophilus that included a low-, mid-, and high-EPS producing strain. Again, we showed significant improvements in EPS isolation using a proteolytic step. In the search for new S. thermophilus strains with enhanced EPS production, accurate quantification in an optimal medium is essential.

## INTRODUCTION

Bacterial fermentation of dairy products is not a recent application and has been widely used in many cultures for thousands of years (1, 2). Rather than relying on natural ferments, companies now ‘design’ fermented food products with targeted favorable characteristics by changing the bacterial composition or culture conditions of the starter strains. In yogurt production, lactic acid bacteria (LAB) are widely used, and specific consortia are selected that will impart the desired product characteristics, such as flavor and textural properties, and aid in food safety through product acidification. The specific composition of such consortia is determined through experience, employing these cultures in fermentations and laboratory-based tools to measure the production of specific metabolites mediating desirable outcomes, such as increased proteolytic ability to increase gut-digestibility (3, 4), faster acidification rates to enhance food safety (5), production of flavor compounds (6), and changes in rheological properties (7), such as texture, through the production of extracellular polysaccharide (EPS). These characteristics are imparted to the product through the metabolic processes of the bacteria during fermentation without the need for food additives. By convention, a yogurt must contain Streptococcus thermophilus and Lactobacillus delbrueckii subsp. *bulgaricus.* Both strains synergistically benefit from their complex interactions with each other (8), and S. thermophilus is predominantly associated with EPS production to enhance textural properties of yogurt.

As the name implies, EPS is a polymeric polysaccharide produced by a bacterium, and is either attached to the outer surface (capsular polysaccharide) or secreted into the extracellular milieu (free-EPS). For S. thermophilus, EPS can range in size from 10 to 2,000 kDa. Its chemical and structural composition is strain-dependent and most commonly comprises repeating units of d-galactose, d-glucose, l-rhamnose, and *N*-acetylgalactosamine (9, 10). Strains that produce free-EPS increase the viscosity of the medium and are called ‘ropy’. These are the preferred choice in commercial applications, such as in yogurt production, where they enhance the textural properties of the product, such as the creaminess and ‘mouthfeel’ (11). Different strains of S. thermophilus have been assessed for their ability to produce EPS, with the highest EPS-producers also increasing the apparent viscosity the most (12, 13). Strains producing over 150 mg/L EPS are considered to be high-producing strains, with Zisu and Shah (14) able to modify culture conditions to yield >1,000 mg/L using S. thermophilus 1275.

EPS concentration is quantified using the widely applied phenol-sulfuric acid method for quantification of sugars (15), after samples are purified to remove contaminating compounds from the growth medium. The first step involves either the removal of proteins through trichloroacetic acid (TCA) precipitation (13) or the precipitation of the EPS, away from the proteins, using cold ethanol (16–18). However, EPS is a large polymer, able to interact with many different components in the growth medium or fermentation substrate, such as casein micelles or whey proteins in fermented milk (19). Therefore, EPS may be removed from the sample coincident to protein removal. This would lead to underestimation of the actual amount of EPS produced by strains of interest. To address the problem of residual protein content interfering with EPS measurements, Zisu and Shah (14) employed an additional measure by adding a proteinase step; however, as TCA had already been used to precipitate proteins, any bound EPS would have already been lost at this stage. Pintado et al. (20), Gancel and Novel (21), and Kimmel and Roberts (22) performed proteolysis as a first step; however, none of these studies were performed on milk or fermented milk substrates.

Therefore, it was thought that a proteolysis step as a first stage of EPS extraction when using either milk or fermented milk may increase extraction yield and prevent loss due to protein interactions. The method was compared to two widely used standard protocols: one in which proteins are initially precipitated through the addition of TCA (Method A), and a second in which EPS is initially precipitated using cold ethanol (Method B). A variety of common bacterial growth media were used, including a chemically defined medium (CDM) developed for S. thermophilus; a complex medium, M17; and reconstituted skim milk (RSM). CDM, M17 and RSM represent media free of large proteins, typical bacterial growth medium conditions, and real-world application conditions, respectively. The protocols were further evaluated in the context of three different commercial S. thermophilus strains known to produce yogurts with different levels of gel-firmness and mouthfeel, one strain unknown but thought to be low, one mid-range strain, and one high-range strain.

## RESULTS

### Xanthan gum-spiked samples.

**FIG 1:**
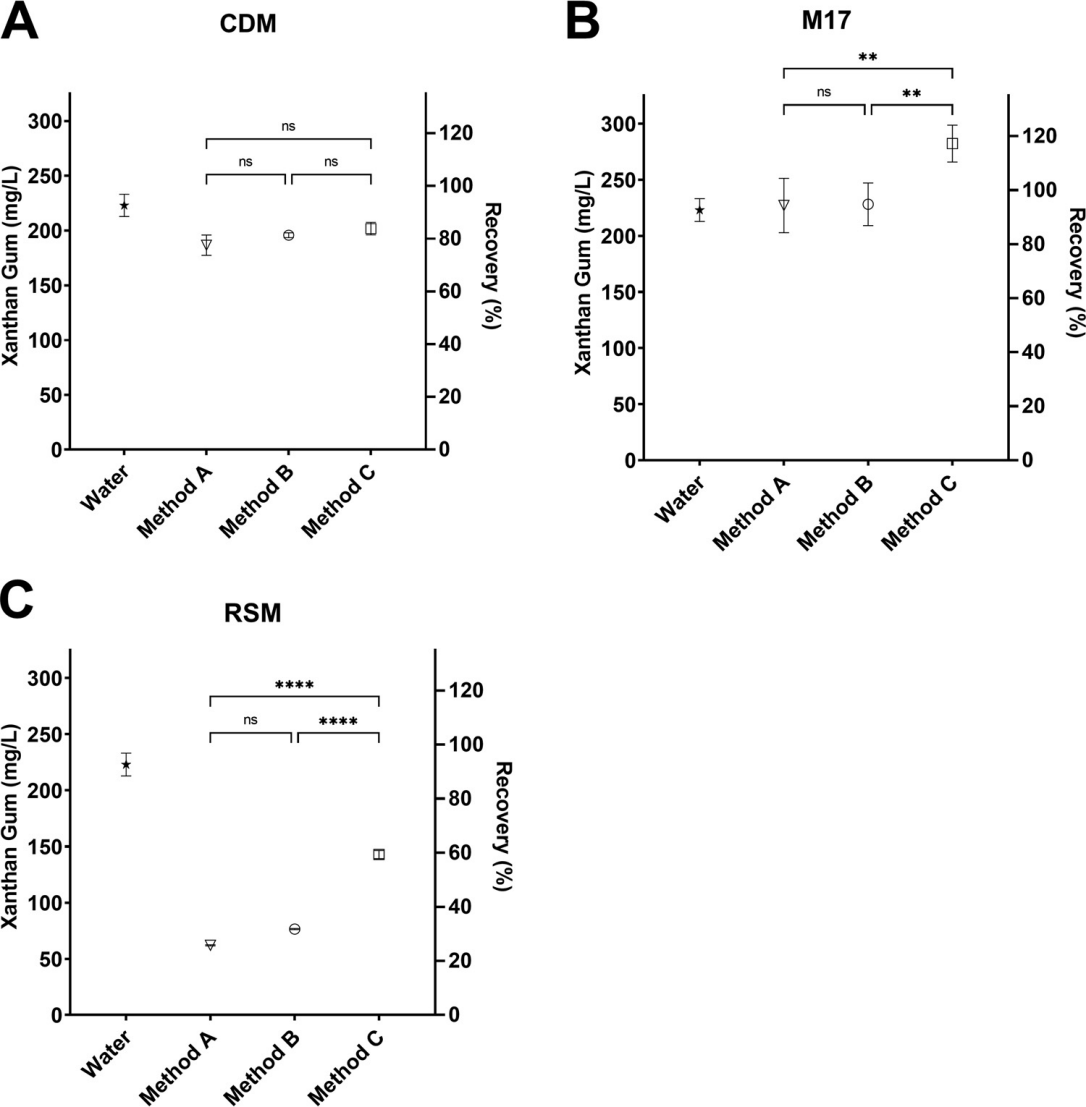
The recovery of xanthan gum was evaluated using three isolation protocols after it had been spiked into (A) CDM, (B) M17, or (C) RSM at a concentration of 225 mg/L. Xanthan gum added directly to water was used as the reference concentration. Samples were analyzed using a one-way analysis of variance (ANOVA) with Tukey’s multiple-comparison test; ns, not significant; **, *P* < 0.01; ****, *P* < 0.0001.

Xanthan gum was spiked into CDM (Fig. 1A), M17 (Fig. 1B), or RSM (Fig. 1C) at a concentration of 225 mg/L, extracted using the three methodologies, and quantified. Xanthan gum spiked directly into water and quantified without going through the extraction procedure was used as a reference. Figure 1A demonstrates that the recovery of xanthan gum from CDM was similar across all three methodologies, with no significant differences observed. On average, Method A had a recovery rate of 83.7 ± 3.8%, followed by Method B with a recovery rate of 87.8 ± 0.9%, and Method C was the best, with a recovery rate of 90.5 ± 2.1%. M17 is a common rich medium used in the cultivation of S. thermophilus. Xanthan gum spiked into M17 resulted in efficient recovery using methods A and B, with no significant difference observed (Fig. 1B). Method C achieved a significantly higher recovery than methods A and B did (Fig. 1B; *P* < 0.01). During analysis, it was determined that M17 medium without the addition of xanthan gum resulted in very high background signal, measuring an average of 210.9 mg/L EPS for all three methodologies, compared to CDM and RSM (Fig. S2 in the supplemental material). Many studies have analyzed the composition of different yeast extracts and demonstrated the high level of batch variability and the presence of many polysaccharides and sugar-peptide complexes (23–25). It is possible that these contributed to some variation in EPS concentration when samples were subjected to protease treatment in Method C. However, the best medium to assess EPS production is RSM, as this most closely resembles the actual composition of milk used during yogurt production. Figure 1C demonstrated that in RSM, methods A and B recovered similar levels of xanthan gum at 62 (27.8%) and 76.5 mg/L (34.3%), respectively, with no significant difference observed. With the addition of a protease step, Method C recovered significantly more xanthan gum than the previous methods at 142.8 mg/L, or 64% (Fig. 1C; *P* < 0.0001). This demonstrated that a large proportion of the xanthan gum is lost during sample processing when using methods A and B. Method C showed significant improvements in xanthan gum extraction in proteinaceous samples, and therefore warranted further assessment using bacterial samples.

### EPS isolation from samples exposed to bacterial growth.

**FIG 2:**
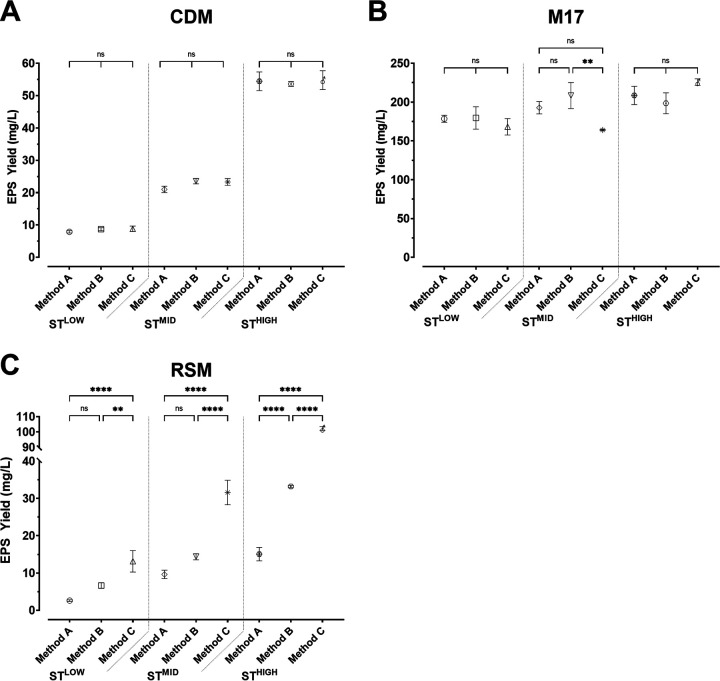
EPS produced by S. thermophilus strains ST^LOW^, ST^MID^, and ST^HIGH^ grown in (A) CDM, (B) M17, and (C) RSM was isolated using three methodologies. Samples were analyzed using a one-way ANOVA with Tukey’s multiple-comparison test; ns, not significant; **, *P* < 0.01; ****, *P* < 0.0001.

When grown in CDM, ST^LOW^ produced the smallest amount of EPS, >10 mg/L (Fig. 2A). ST^MID^ grown in CDM produced 21.0 to 23.5 mg/L EPS, approximately 2-fold more than ST^LOW^ (Fig. 2A); and ST^HIGH^ produced a further 2-fold increase in EPS, with an average of 54 mg/L. Consistently, for each strain employed, there were no significant differences in the amounts of EPS isolated by any of the methods tested.

Figure 2B shows that when strains were grown in M17, the amount of EPS produced was similar across all three bacteria. ST^MID^ exhibited a significant decrease in EPS isolated using Method C compared to Method B (Fig. 2B; *P* < 0.01); however, this was the only difference observed within each bacterial sample type. When M17 samples were clustered based on the method used (Fig. S3), there were no significant differences observed using either method A or B; however, there was a significant increase in the amount of EPS isolated for ST^HIGH^ compared with ST^LOW^ or ST^MID^ (*P* < 0.0001) when using Method C. Based on previous results (Fig. S2), the purported high amount of EPS produced is most likely an artifact of the medium due to the high background signal caused by different medium components. Despite normalizing the EPS concentration by removing the background contamination, the amount of EPS produced does not reasonably match that in other media with the same bacterium.

When ST^LOW^ was grown in RSM, there was no significant difference between methods A or B (Fig. 2C). When using method C, there was a significant increase in the amount of EPS extracted compared with both methods A (*P* < 0.0001) and B (*P* < 0.01), with 13.2 mg/L EPS measured. This observation was mirrored for ST^MID^, with no significant difference observed between methods A (9.64 mg/L) and B (14.4 mg/L); while Method C isolated 31.6 mg/L, significantly more than either method A or B (*P* < 0.0001). As expected, ST^HIGH^ yielded the highest concentration of EPS isolated using each method. Method A isolated 15.1 mg/L EPS, while Method B demonstrated a greater-than-2-fold improvement in EPS yield (33.2 mg/L; *P* < 0.0001). This was further enhanced with another 3-fold improvement over Method B when using Method C, isolating 102.2 mg/L EPS from ST^HIGH^ (Fig. 2C; *P* < 0.0001). In summary, there was a general trend where Method B had a slight improvement over Method A in RSM, which was significant when using higher EPS-producing bacteria. However, although methods A, B, and C performed equally well in CDM, Method C clearly outperformed both standard protocols in RSM.

## DISCUSSION

Identifying and developing new starter strains with increased EPS production is not only of industrial relevance for fermented dairy products, but can also be applied to many different products for modifying rheological properties. Therefore, it is of the utmost importance to use a methodology which is appropriate for the sample type, reliable, and accurate. When using CDMs, such as the one developed in this study, all methodologies performed equally well using artificial samples, those spiked with xanthan gum, and those with bacteria-produced EPS. This was expected, as CDM does not contain any large peptides or extraneous media components that may hinder EPS extraction or cause background signal during analysis. Although CDMs are excellent for controlling the exact medium composition, not all S. thermophilus strains grow equally well in them, and thus CDM is generally not the ideal screening medium for comparing EPS production in multiple strains without first assessing their growth dynamics. However, when CDM enables sufficient bacterial growth, it provides a good environment for the accurate measurement of basal EPS production, where the specifics of isolation methods become less relevant. CDM also allows for the control of specific nutrients, such as carbon and nitrogen sources, so that their respective effects on EPS production can be investigated (26).

During analysis, it was observed that different batches of M17 resulted in varying levels of background signal (data not shown), leading to high variability in the quantification of EPS samples. Even with background correction, the measured EPS concentrations did not correspond well with previous data which showed ST^LOW^ to be a low EPS-producer. Further analysis revealed yeast extract to be the source of this variation (data not shown). Batch variation in different yeast extracts has been documented previously (23–25), and the presence of large peptide-sugar complexes such as mannoproteins may be a source of error when assessing EPS production in a medium with these components, as similarly described by Kimmel and Roberts (22). Gorret et al. (27) observed with Propionibacterium acidipropionici that increasing yeast extract concentration was linearly correlated with an increase in EPS production, attributed to increased growth of the bacterium. The possibility cannot be excluded that M17 medium may have promoted EPS production in the bacteria tested beyond that which was observed in CDM and RSM. However, one would expect the trends to be maintained when observed across the other mediums, which they were not. This observation does warrant some consideration when choosing the appropriate growth medium for an assay, and hesitation in reported EPS concentrations when using these types of rich medium.

Rimada and Abraham (28) performed detailed analyses of 16 different EPS extraction methods. It was determined that heat-treating milk samples as a first step in EPS isolation and dialysis to remove residual sugars (i.e., lactose) from the medium is essential. All the methodologies presented in the current study incorporated these steps. Rimada and Abraham (28) highlighted 4 of the 16 methodologies which yielded the best results; one of these, used in this study, closely resembles Method B. We tried to further improve on this method by incorporating a proteolytic step after heat treatment. When isolating xanthan gum from RSM, reductions in recovery were observed even in the absence of bacterial fermentation which would modify the milk matrix and further alter the rheological properties. This was significantly improved through the addition of a proteolysis step, suggesting there are interactions between the EPS molecules and the casein micelles and/or whey proteins in milk and that these interactions hindered xanthan gum recovery yields. These interactions have been observed microscopically through electron microscopy techniques (29, 30) as well as by confocal scanning laser microscopy (19). The interactions between EPS and milk proteins contribute to the microstructure of fermented milks and are favored for improving the rheological and physical properties of the final products. However, these interactions present challenges for qualifying the amount of EPS in these products. When using a proteolytic step prior to EPS precipitation (Method C), significant increases in EPS extraction were possible for all three bacteria tested, with the most notable improvement for the highest EPS-producing strain, which isolated more than 6.5-fold more EPS compared to Method A. Zisu et al. (14) incorporated a proteolysis step into their isolation procedure; however, this step was after ethanol precipitation, as in Method B of this study, which demonstrated that a large amount of the EPS had already been lost. Kimmel et al. (31) and Gancel and Novel (21) both implemented a pronase step into their EPS isolation methodology; however, these extractions were not performed in milk. We demonstrated that incorporating a protease step prior to EPS precipitation resulted in significant improvements in EPS isolation when using milk as a growth medium, while other matrices, such as CDM, which lacked the complex structure of milk showed no significant improvements in any of the methodologies.

As textural properties are a key factor in consumer preference of fermented milk products, the screening of bacteria that impart these traits must be performed accurately. It is possible that promising bacterial strains have been rejected for commercial use based on inaccurate screening data. Originally, the strains employed in this study were chosen based on differences in the perceived mouth-feel and thickness of yogurt they produce, factors that are known to be influenced by EPS production (32). Strain ST^HIGH^ achieves the highest rating in these attributes and, in this study, produced the most EPS. Similarly, ST^MID^ is known to be mid-range in both attributes and produced a mid-range amount of EPS, while ST^LOW^ produced the smallest amount of EPS. Although studies have not been performed on ST^LOW^ to determine the perceived mouth-feel and thickness of its yogurt product, it could be hypothesized, based on these findings, that the resultant product would score low in these factors. Accurate EPS quantification is important for differentiating between strains which cover the continuous spectrum between the extremes of these attributes. This will be important for the development of new starter strain consortia and for bioprospecting new starter strains for EPS production.

## MATERIALS AND METHODS

### Optimization of conditions for EPS isolation.

To assess the efficiency of EPS in high protein versus low protein sample types, xanthan gum (IngredientStop, Auckland, NZ) was initially added to sterile medium without bacteria for the development of the methods. Xanthan gum was added to a final concentration of 225 mg/L to M17 medium (tryptone 5 g/L, soya peptone 5 g/L, Lab-LEMCO 5 g/L, yeast extract 2.5 g/L, ascorbic acid 0.5 g/L, MgSO4 0.25 g/L, di-sodium-glycerophosphate 19 g/L [pH 6.9]) with 0.5% (wt/vol) sucrose, a chemically defined medium (CDM) (lactose 10 g/L, sodium acetate 1.0 g/L, triammonium citrate 0.6 g/L, KH_2_PO_4_ 3.0 g/L, K_2_HPO_4_ 3.0 g/L, urea 0.24 g/L, ascorbic acid 0.5 g/L, MgCl_2_·6H_2_O 0.2 g/L, CaCl_2_·2H_2_O 0.05 g/L, pyridoxamine-HCl 0.005 g/L, nicotinic acid 0.001 g/L, riboflavin 0.0004 g/L, calcium pantothenate 0.001 g/L, thiamine-HCl 0.001 g/L, 2 mM amino acid mixture (alanine, arginine, aspartate, cysteine, glutamate, glutamine, glycine, histidine, isoleucine, leucine, lysine, methionine, phenylalanine, proline, serine, threonine, tyrosine, and valine [pH 6.5]), or 9.1% (wt/vol) reconstituted skim milk (RSM) sterilized at 110°C for 10 min. Samples were incubated at 37°C for 24 h to mimic bacterial growth conditions and further processed for EPS isolation.

### Isolation of EPS.

Three isolation methods were compared; details for each method are outlined below and a schematic is shown in Fig. S1 in the supplemental material.

**(i) Method A.** Method A uses a protocol similar to that of De Vuyst et al. (13) where protein is initially precipitated, with a few minor modifications. After incubation at 37°C for 24 h, 5-mL samples were heated at 95°C for 15 min to inactivate endogenous bacterial enzymes in the growth medium. Trichloroacetic acid (TCA) was added to a final concentration of 20% (vol/vol) and the samples were incubated for 8 h at 4°C to precipitate proteins. The precipitate was removed by centrifugation at 12,000 × *g* for 15 min at 4°C. The supernatant was collected, and anhydrous ethanol was added to the supernatant at a ratio of 3:1 vol/vol, then stored at 4°C overnight. Samples were centrifuged at 12,000 × *g* for 20 min at 4°C to collect the pellet containing EPS. The pellet was dissolved in 2 mL sterile ultrapure water and dialyzed in 8- to 12-kDa dialysis tubing placed in 4 L water for 48 h, with the water changed every 12 h. After dialysis, samples were transferred into 2-mL centrifuge tubes, and the insoluble fraction was removed by centrifugation at 10,000 × *g* for 10 min at 4°C. The supernatant could then be quantified, as described below.

**(ii) Method B.** Method B follows the protocol used by Zhang et al. (16) where EPS is initially precipitated, with a few minor modifications. After incubation at 37°C for 24 h, 5-mL samples were heated at 95°C for 15 min to inactivate endogenous bacterial enzymes in the growth medium. Three volumes of anhydrous ethanol were added to the sample, which was placed at 4°C overnight and centrifuged at 12,000 × *g* for 15 min at 4°C. The supernatant was discarded, and the pellet was dissolved in 5 mL sterile ultrapure water. TCA was added to the sample solution to a final concentration of 20% (vol/vol, and the sample was placed at 4°C for 8 h. Samples were then further processed from a similar stage to that described in Method A.

**(iii) Method C.** Method C follows protocols similar to those of Pintado et al. (20) with a few modifications. Five-milliliter samples were adjusted to a pH of 7, heated at 95°C for 15 min to inactivate any enzymatic activity in the samples, and then cooled down to 55°C. Five units of proteinase K (50 U/mL, Macherey-Nagel GmbH & Co. KG, Germany) were added, and the sample was incubated at 55°C for 16 h. After incubation, samples were processed as in Method B, beginning with the initial ethanol precipitation step.

### Quantification of EPS.

To determine the concentration of EPS in each sample, the standard phenol-sulfuric acid method was used (15, 33). After EPS had been isolated as described above, an aliquot of the sample (100 μL) was mixed 1:1 with 6% (vol/vol) phenol, and 1 mL concentrated sulfuric acid was quickly added afterwards. The sample was mixed well and incubated at room temperature for 30 min. Absorbance at 480 nm was used to analyze the colorimetric reaction. A standard curve was generated for each individual experiment, using solutions of known glucose concentrations. The concentration of EPS in each sample was calculated based on the comparison to the standard curve, taking into account any dilution of the sample, as well as the initial volume and the final volume remaining after dialysis. Xanthan gum, added directly to sterile ultrapure water at 225 mg/L, was used to assess the accuracy of the standard curve generated. Samples of each respective medium were used to control for background signal.

### Bacterial strains and growth conditions.

To assess the isolation of EPS from samples fermented with bacteria, a low EPS-producing strain was received from Fonterra Co-Operative Group Ltd. (S. thermophilus ST^LOW^). Two other commercial cultures used in yogurt production were obtained (CHR-Hansen), YoFlex YF-L811 and Premium 5.0. Both commercial cultures are a combination of S. thermophilus and *L. delbrueckii* subsp. *bulgaricus*. Each S. thermophilus was isolated from the coculture. Samples were grown in M17 medium supplemented with 1% (wt/vol) lactose at 37°C and plated onto M17 agar. Multiple colonies were selected and used to inoculate M17 medium. This process was repeated for three generations (34). Successful isolation of S. thermophilus was assessed microscopically to ensure that an axenic culture was obtained prior to further experiments. YoFlex YF-L811 produces a yogurt with a mid-range mouth-thickness score, and YoFlex Premium 5.0 produces a yogurt with a high-range mouth-thickness score, here referred to as ST^MID^ and ST^HIGH^, respectively.

When required, bacteria were propagated on M17 medium for 2 generations before use. Bacteria were centrifuged at 10,000 × *g* for 1 min at room temperature, resuspended in phosphate-buffered saline (pH 7.4), and adjusted to an optical density at 595 nm (OD_595_) of 1.0. This was used to inoculate 9.1% RSM, CDM, or M17 medium using a 2% (vol/vol) inoculum. Samples were incubated at 37°C for 24 h prior to EPS isolation as described above.

### Statistical analysis.

Experiments were minimally performed in biological triplicates, each with at least two technical replicates. Data were analyzed using GraphPad Prism (version 9.0.0 for Windows, GraphPad Software, San Diego, CA, USA) using a one-way analysis of variance with either the Dunnett’s or Tukey’s *post hoc* multiple-comparison test, as described in the figure legends.
