# Supplementary material for: Extracellular Polysaccharide Extraction from Streptococcus thermophilus in Fermented Milk
Source: Microbiol Spectr. 2022 Mar 28;10(2):e02280-21. doi: 10.1128/spectrum.02280-21 (PMC9045140; doi:10.1128/spectrum.02280-21)
Supplement: SUPPLEMENTAL FILE 1 — Supplemental material. Download SPECTRUM02280-21_Supp_1_seq6.pdf, PDF file, 0.3 MB [file spectrum02280-21_supp_1_seq6.pdf]

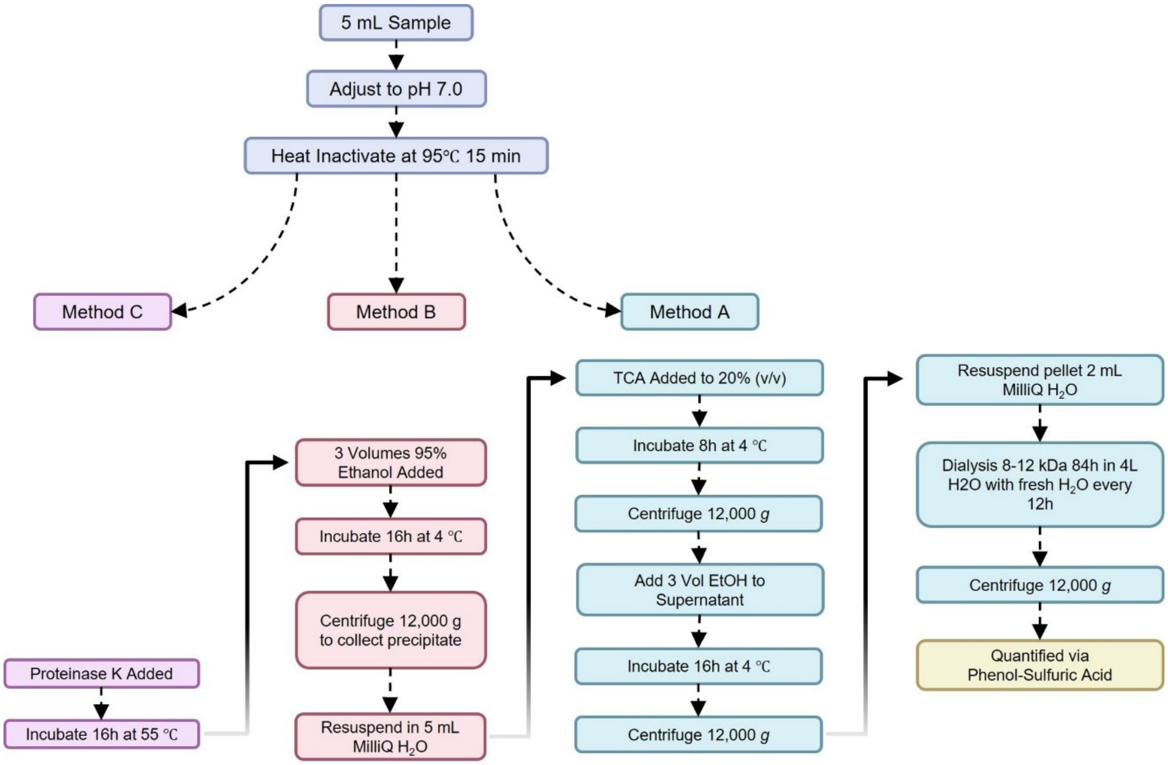

Supplemental Figure 1. Schematic representation of the three isolation procedures used.

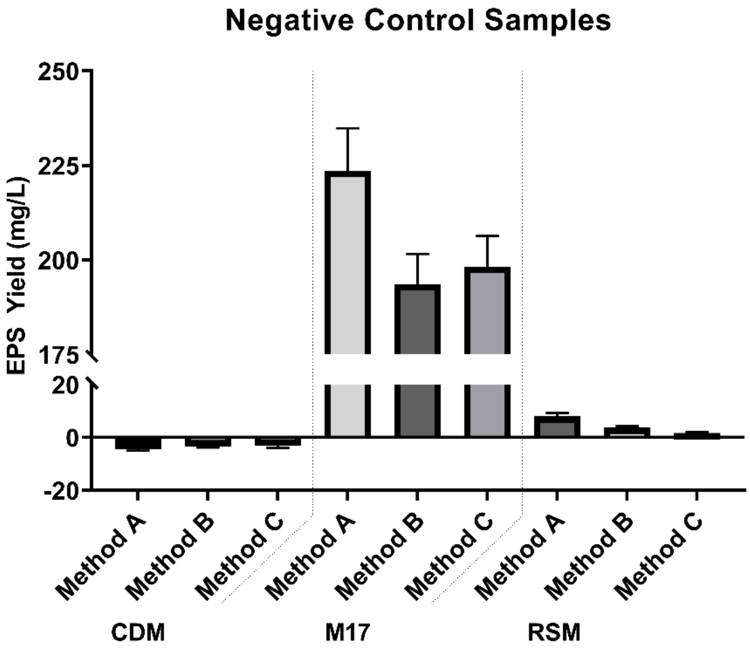

Supplemental Figure 2. Background concentration of EPS measured in CDM, M17 and RSM media without the addition of xanthan gum or bacteria.

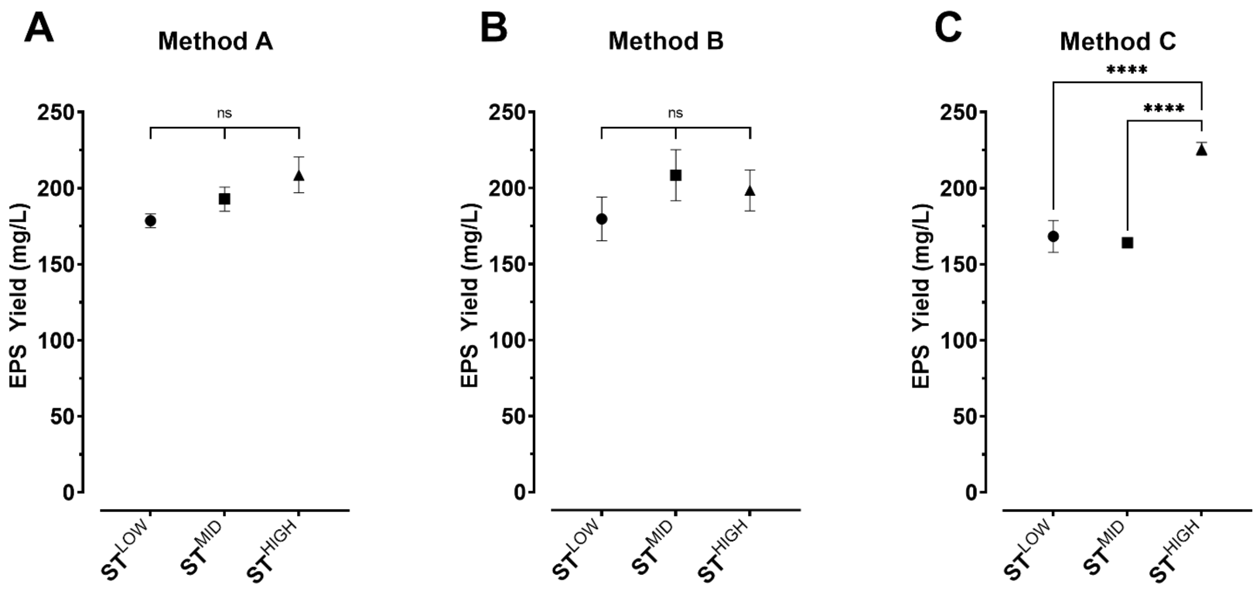

Supplemental Figure 3. Concentration of EPS isolated from *S. thermophilus* ST^{LOW}, ST^{MID} or ST^{HIGH} grown in M17 medium using three isolation procedures. Samples were analyzed using a one-way ANOVA with Tukey's multiple comparison test; ns – not significant, ****p<0.0001.
